# Moving Research to Practice: A Qualitative Study Exploring Patient Perspectives of the Implementation and Sustainability of a Digital Health Intervention for Chronic Kidney Disease

**DOI:** 10.2196/91966

**Published:** 2026-06-30

**Authors:** Courtney J Lightfoot, Roseanne E Billany, Gurneet K Sohansoha, Thomas J Wilkinson, Melanie J Davies, Thomas Yates, Alice C Smith, Matthew PM Graham-Brown

**Affiliations:** 1 Division of Cardiovascular Sciences, University of Leicester, Leicester, England, United Kingdom; 2 Leicester Partnership for Kidney Health Research, University of Leicester, University Road, Leicester, England, LE1 7RH, United Kingdom, 44 116 258 4346; 3 NIHR Leicester Biomedical Research Centre, Leicester, England, United Kingdom; 4 Diabetes Research Centre, University of Leicester, Leicester, England, United Kingdom; 5 Division of Global, Lifestyle and Metabolic Health, University of Leicester, Leicester, England, United Kingdom; 6 Department of Renal Medicine, University Hospitals of Leicester NHS Trust, Leicester, England, United Kingdom

**Keywords:** chronic kidney disease, digital health, implementation, implementation science, self-management, patient education, health education, awareness

## Abstract

**Background:**

My Kidneys & Me (MK&M) is a digital health intervention (DHI) codeveloped to provide specialist health and lifestyle education aimed at enhancing self-management among individuals with chronic kidney disease. The MK&M DHI was shown to improve self-management behaviors in a multicenter randomized controlled trial (SMILE-K), but strategies to support effective integration into routine clinical practice are not known.

**Objective:**

This study aimed to explore patient perspectives about real-world implementation and sustainability of the MK&M DHI.

**Methods:**

For this substudy, participants from the SMILE-K trial were invited to take part in a semistructured qualitative interview to explore their perspectives on the implementation and sustainability of the MK&M DHI in routine clinical practice. Barriers and facilitators that could potentially influence the uptake and usage of the program in real-world settings, outside of a clinical trial, were also explored. Topics included accessibility, usability, integration into routine care, and types of support required to sustain engagement over time. Data were analyzed using reflexive thematic analysis.

**Results:**

A total of 42 interviews were conducted with 35 individuals (mean age 62.5, SD 9.9, range 38‐84 years; 23/35, 66% male; 33/35, 94% White British; estimated glomerular filtration rate: mean 33.5, SD 12.4 mL/min/1.73 m^2^). Five themes were identified, encompassing a range of critical factors that should be addressed to ensure the successful implementation of MK&M into routine kidney care and its alignment with real-world clinical needs. These include ensuring that information is timely, relevant, and tailored to individual needs. Participants highlighted the importance of promotion through multimodal strategies and approaches, leveraging diverse communication channels to maximize reach and engagement. Equitable access was identified as a priority, with potential barriers, such as digital literacy and digital poverty, requiring attention. Building trust, through reassurance and endorsement from trusted health care professionals, was seen as essential to strengthen confidence in MK&M. Finally, participants stressed the need for continued engagement and sustainability, ensuring that MK&M is successfully integrated into care pathways and supported over time to maintain its impact.

**Conclusions:**

The study findings highlight that timely, well-targeted communication using diverse strategies will be critical for the successful uptake of MK&M among people living with chronic kidney disease. The identification of perceived factors that will influence implementation of the program provides actionable insights to guide the development of tailored implementation strategies to ensure that MK&M is relevant, acceptable, and feasible in real-world practice. These findings provide a foundation for designing approaches that are not only patient-centered and inclusive but also adaptable to diverse clinical contexts. By addressing these factors proactively, implementation efforts can promote equitable access, foster sustained engagement, and support the long-term integration of DHIs, such as MK&M, into routine kidney care.

## Introduction

Chronic kidney disease (CKD) affects approximately 13% of the general population worldwide [1]; it is currently the 10th leading cause of death, and projected to be the fifth leading cause of premature death worldwide by 2040 [2,3]. Individuals with CKD face a significantly higher risk of morbidity and mortality, primarily due to associated cardiovascular disease [4], and live with poor health and high symptom burden [5]. Self-management plays a critical role in helping people with CKD monitor their health, adhere to treatment plans, maintain a healthy lifestyle, and make informed decisions about their care [6,7]. Engaging in effective self-management behaviors can slow progression of the disease, reduce the risks of associated complications, and improve quality of life [6,8]. Providing accessible education, practical strategies, and interactive resources can empower patients to take greater control of their condition and stay engaged in their health care.

To support people living with CKD to better self-manage their condition, we developed a digital health intervention (DHI) called “My Kidneys & Me” (MK&M) [9]. MK&M is an evidence- and theory-based DHI designed to deliver comprehensive CKD-specific self-management education, support, and guidance for individuals with mild to moderate CKD [9], and is hosted on the MyDESMOND (a diabetes digital self-management program) platform [10]. The MK&M program was codeveloped with kidney health professionals, researchers, digital health specialists, and experts in complex intervention design, in partnership with people living with CKD and their families. MK&M offers a structured, interactive, and tailored approach to improve (1) patient activation, which includes enhancing awareness, understanding of CKD, health-related knowledge, confidence, and skills for engaging in health-promoting behaviors; and (2) CKD self-management behaviors, including symptom management, lifestyle changes, and physical function [9,11,12]. The development of MK&M and its features is discussed in detail elsewhere [9,11]. The MK&M DHI has been well received and used by people with CKD [13] and has demonstrated positive effects, particularly among those with low levels of activation (knowledge, skills, and confidence in managing their own health) and among those who used the program more [12].

Having demonstrated efficacy in a large multicenter randomized controlled trial, the next critical step is to understand how best to implement MK&M in routine clinical practice to maximize its impact on patient care and health outcomes. Translating research findings into clinical practice is essential to ensure that evidence-based interventions deliver real-world benefits for patients [14]. Successful implementation requires collaboration with health care professionals, integration into existing care pathways, and consideration of patient needs, including accessibility and usability [15,16]. Actively incorporating patient perspectives on how to improve access, engagement, and use of DHIs, such as MK&M, in routine clinical practice is essential to ensure successful implementation and that the program reflects real-world needs. Understanding key barriers and facilitators can further inform the development of targeted implementation strategies that enhance the efficiency of intervention delivery and support its successful uptake and large-scale adoption. Thus, the aim of this study was to explore patients’ perspectives on the real-world implementation and sustainability of the MK&M DHI.

## Methods

### Study Design

This qualitative substudy comprises qualitative data collected as part of the SMILE-K trial (ISRCTN18314195). The SMILE-K study protocol [9], pilot study [16], and primary findings [10,11] are published elsewhere. As this paper explores participants’ perceptions of the implementation and sustainability of the MK&M DHI, the data presented are predominantly from those who had access to and used the program (intervention group); data from the control group are included but are limited. The COREQ (Consolidated Criteria for Reporting Qualitative Research) guidelines were followed when reporting this study to enhance rigor, transparency, and completeness [17].

### SMILE-K Study Design

The SMILE-K trial was a 20-week prospective, mixed methods, multicenter, randomized controlled, and parallel-group trial recruiting patients from 26 hospital sites across England between May 2021 and December 2022. All study procedures were conducted remotely due to the COVID-19 pandemic and to facilitate pragmatic evaluation. The study used a parallel mixed methods design; quantitative and qualitative data were collected concurrently during the randomized controlled trial. Full details of study methodology are published elsewhere [11,18]. Randomization was conducted using a computer-generated sequence in a 2:1 ratio (intervention:control), stratified by age (≤63 years vs >63 years). Group allocation was not blinded to researchers at the lead site or participants; however, researchers at local sites were blinded. The intervention group received the MK&M program, while the control group continued usual care, maintaining their lifestyle and clinical care plans. Control participants did not access MK&M during the trial but were offered the program upon study completion.

### MK&M Intervention

The MK&M program is a web-based application, hosted on the MyDESMOND platform [10], that was systematically developed as a theory- and evidence-based digital self-management intervention for people with nondialysis CKD; it is designed to provide holistic CKD-specific self-management education, support, and guidance for people living with mild to moderate CKD. The development of MK&M and its features is discussed in detail elsewhere [9,11].

The MK&M program delivers content and resources aimed at increasing CKD knowledge and patient activation, reducing health risks, managing symptoms, and improving physical function. Key components include educational “Learn about” sessions covering kidneys, CKD, treatment, symptoms, associated risks, and lifestyle factors (eg, diet and physical activity), alongside behavior-focused “How to...” sessions offering practical strategies for making small, sustainable health and lifestyle changes. These sessions support goal setting, self-monitoring, and action planning to help individuals apply learning. Additional features include health trackers (eg, blood pressure, symptoms, and exercise), integration with activity devices (Fitbit, Garmin, and Google Fit), goal-setting tools, and social support forums. Content is delivered in varied formats, including text, graphics, animations, and videos, alongside interactive quizzes to reinforce knowledge.

For the SMILE-K trial, new content was released weekly for 10 weeks, so 20 weeks was chosen as the primary end point to allow participants time to review the educational content, put learning into action, and explore other features on the program. As part of the MK&M program, participants received automated email reminders when new content (such as “How to...” sessions) became available. Additional reminders were sent if participants had not logged in after 7, 14, or 28 days. While the program can send push notifications, these were disabled for the study to allow participants to use the program as they wished. Participants’ usability and experiences of using the program have been described elsewhere [13].

### Participants and Recruitment

To participate in the main trial, participants had to be aged 18 years and older with CKD stages 3-4 (estimated glomerular filtration rate [eGFR] 15‐59 mL/min/1.73 m^2^) or diagnosed with a progressive kidney condition with an eGFR >60 mL/min/1.73 m^2^ (eg, polycystic kidney disease), not receiving renal replacement therapy. Eligible individuals were approached via an invitation letter and promotional flyer detailing the SMILE-K study and MK&M DHI by a member of their clinical team or a research nurse. Those interested contacted the research team by email to receive a web-based information sheet and electronic consent form. The information sheet detailed the data collected using the MK&M program and copies of the MyDESMOND (platform on which MK&M sits) ‘Terms of Use,” “Privacy/Cookies Policy,” and “Acceptable Use Policy.” After providing informed consent, participants completed an online baseline survey that collected outcome measures (detailed elsewhere [11]) via a secure link (Jisc Online Surveys). Following survey completion, participants were randomized (2:1) to either the intervention or control group. Participants randomized to the intervention group received immediate access to the MK&M program via a direct link with a unique user log-in. The control group participants were provided access following study completion. A video orientation to the platform was available upon login to MK&M, and technical support was provided to those who requested it.

All trial participants were eligible for the qualitative substudy. Interview participants were purposively selected to capture a diverse range of participant characteristics to ensure a representative population. Potential participants were sent an invitation letter via email, with a web-based information sheet and electronic consent form. Participants were contacted via email to arrange a suitable date and time for their interview. Participants were recruited to baseline interviews between November 2021 and October 2022, with postintervention interviews conducted between February 2022 and March 2023. All participants were approached at 10 weeks; a 20-week interview was offered to those who had completed the 10-week interview and agreed to further follow-up, had missed or declined the 10-week interview but remained willing to participate later, or if inclusion at 20 weeks supported maximum variation sampling. The control group participants were offered only 1 interview; intervention group participants could complete 1 or both interviews based on availability and sampling needs. Nonresponders were recontacted once, with no more than 3 total contact attempts across both time points.

### Data Collection

Individual semistructured interviews were used to explore patient participants’ perceptions of the implementation and sustainability of the MK&M DHI in routine clinical practice and barriers and facilitators that could potentially influence the uptake and usage of the program in real-world settings, outside of a clinical trial (eg, SMILE-K). The interview schedule was informed by current literature and developed in consultation with patient and public involvement partners. Topics included accessibility, usability, integration into routine care, and types of support required to sustain engagement over time. A common core interview guide was used across arms and time points to ensure comparability, with intervention-specific probes (eg, intervention engagement/usability and sustainability effects). Although the control group participants had not used the MK&M DHI, they were aware of it, and their perspectives were sought to explore anticipated barriers, contextual influences, and system-level factors relevant to implementation.

For this qualitative substudy, a target sample of 25‐35 participants (20‐30 intervention group and 5‐10 control group) was set based on the concept of information power [19] and resource availability. Purposive sampling was used to capture a diverse range of perspectives. The adequacy of the sample size was continuously reviewed during data collection to ensure that an in-depth understanding of the investigated phenomena was obtained.

Participants were interviewed by telephone at 10 and/or 20 weeks postrandomization, using the same topic guide. Interviews lasted approximately 60 minutes, were audio-recorded, and transcribed verbatim by a professional service. All interviews were conducted by a single researcher (CJL), who is an experienced qualitative researcher. Participants had no relationship with the researcher prior to study commencement. The researcher kept a personal reflective logbook during interview conduction and analysis.

### Data Analysis

All identifiable information, including names and personal details, was removed from transcripts. Data were managed in NVivo (version 12; QRS International) and analyzed using interpretive reflexive thematic analysis following Braun and Clarke’s approach [20]. This provides flexibility and an inductive, meaning-focused analytic process, allowing for interpretation of how participants made sense of their experiences while acknowledging the researchers’ active, reflexive role in theme development. Initially, 1 researcher (CJL) reviewed the full dataset to familiarize themselves with the content through a process of immersion, noting early impressions and potential ideas. They then identified segments of the data that appeared potentially interesting and relevant, generating and applying initial codes, which were refined and finalized through review. Each transcript was independently coded by CJL. A sample of transcripts was checked by another researcher (REB) and cross-referenced against a developed codebook. Codes were compiled into clusters and collated together to form candidate themes. Potential themes were identified by CJL and reviewed and refined with REB through an iterative process, assessing to ensure the fit of the themes in relation to the data, both the coded extracts and the overall dataset. The researchers clearly defined and named the themes to provide detailed descriptions and clarification about what the theme is about and how it contributes to the analytical story. CJL and REB met regularly to discuss and reflect on the analysis and consider alternative interpretations; consensus was achieved through an iterative collaborative approach. A codebook and detailed analytic diary were maintained to ensure consistency and transparency. Findings are presented as direct quotes with participant ID, sex, age, and study group (I: intervention; C: control).

### Reflexivity Statement

Both CJL and REB are experienced, qualitative researchers who had no prior relationship with the participants. CJL has expertise in patients’ experiences of living with and self-managing their long-term condition(s), particularly CKD, as well as in the development, evaluation, and implementation of complex interventions to better support patient self-management. REB has expertise in patients’ experiences of rehabilitation and exercise-based interventions, particularly kidney transplant recipients. While CJL was a codeveloper of the MK&M DHI, the interview participants were not aware of this to reduce any social desirability bias. REB was not involved in the development of MK&M.

To mitigate any potential bias, CJL engaged in reflexive bracketing and kept a reflexive diary, detailing their reflections on their own values and experiences and how these might shape the data. They also met regularly with REB, who was not involved in the development of MK&M, during data collection and analysis to discuss any potential biases that might influence the process and how to prioritize the participants’ voices. Additionally, CJL and REB met with the wider research group to discuss findings and interpretation of the data.

### Ethical Considerations

This study was fully approved by the Research Ethics Committee−Leicester South on November 19, 2020 (reference: 17/EM/0357). All participants provided online informed consent. All participants were given the opportunity to ask questions throughout the consent process. The participants did not receive compensation for participation. The study was conducted in accordance with the Declaration of Helsinki. The trial is sponsored by the University of Leicester (rgosponsor@le.ac.uk). The study was prospectively registered as ISRCTN18314195 on December 18, 2020.

## Results

### Overview

This paper focuses specifically on participants’ views on the implementation and sustainability of MK&M into routine care. Participants’ perspectives and experiences of using MK&M and ongoing engagement are reported elsewhere [13]. No meaningful differences between the groups were identified; thus, findings are reported at an aggregate level to provide a more coherent and analytically robust account of participants’ views.

### Summary of Findings From the SMILE-K Trial

A total of 420 participants were recruited into the SMILE-K trial, of whom 280 (66.7%) participants were randomized into the intervention group and were subsequently provided access to the MK&M program. The SMILE-K trial demonstrated that the MK&M program improved patient activation, although the overall effect was nonsignificant [12]. Notably, the greatest improvements occurred in individuals with low baseline activation and in those who used the program. Furthermore, MK&M was well-received and actively used by the participants [13].

### Participant Characteristics

A total of 55 (36 intervention and 19 control) people were invited to participate, of whom 43 (78%) (31 intervention and 12 control) responded, and 35 (81%) individuals (27 intervention and 8 control) were subsequently interviewed. Interview participant characteristics are displayed in Table 1; in summary, participants had a mean age of 62.5 (SD 9.9; range 38‐84) years, 66% (23/35) were male, 94% (33/35) were White British, and had a mean eGFR of 33.5 (SD 12.4) mL/min/1.73 m^2^. A total of 42 interviews were conducted (29 at 10 weeks [23 intervention and 6 control] and 13 at 20 weeks [10 intervention and 2 control]), with 7 intervention participants interviewed twice at 10 and 20 weeks. Interviews lasted an average of 58 (range 32‐96) minutes.

**Table 1.** Participant characteristics.

Participant ID	Study group	Age (years)	Sex	eGFR^a^	Ethnicity	Education	Employment	Follow-up interviews
SS01^b^	Intervention	38	Female	45.7	White British	University	Y^c^	1‐10 weeks
SS02	Intervention	66	Female	28.4	White British	College	R^d^	1‐10 weeks
SS03	Intervention	71	Female	37.2	White British	Other trade/vocation qualification	Y	1‐10 weeks
SS04	Intervention	45	Female	57.6	White British	High school	Y	1‐10 weeks
SS05	Intervention	61	Female	58.8	White British	Other trade/vocation qualification	R	1‐10 weeks
SS06	Intervention	55	Female	35.9	White British	College	Y	1‐10 weeks
SS07	Intervention	55	Male	20	White British	Other trade/vocation qualification	Y	1‐10 weeks
SS08	Intervention	54	Male	29.8	White British	University	Y	1‐10 weeks
SS09	Intervention	69	Male	24.1	White British	University	R	1‐20 weeks
SS10	Intervention	60	Female	59.2	Other Mixed	College	NR^e^	1‐10 weeks
SS11	Intervention	59	Male	47.5	White British	University	Y	1‐10 weeks
SS12	Intervention	72	Male	33	White British	University	R	1‐10 weeks
SS13	Intervention	57	Female	25.6	White British	University	R	2 times at 10 and 20 weeks
SS14	Intervention	57	Male	35.3	Other White	High school	Y	1‐20 weeks
SS15	Intervention	67	Male	30.1	White British	College	R	2 times at 10 and 20 weeks
SS16	Intervention	68	Male	38.2	White British	College	Y	1‐10 weeks
SS17	Intervention	69	Female	26.9	White British	University	R	2 times at 10 and 20 weeks
SS18	Intervention	60	Male	41.4	White British	High school	R	1‐10 weeks
SS19	Intervention	71	Male	34.5	White British	University	Y=SE^f^	2 times at 10 and 20 weeks
SS20	Intervention	71	Male	35.7	White British	University	Y	1‐10 weeks
SS21	Intervention	71	Male	19.1	White British	University	R	1‐10 weeks
SS22	Intervention	55	Female	23.4	White British	Other trade/vocation qualification	Y	1‐10 weeks
SS23	Intervention	54	Female	21.9	White British	College	Y	2 times at 10 and 20 weeks
SS24	Intervention	72	Male	37.5	White British	High school	R	1‐20 weeks
SS25	Intervention	76	Male	16.7	White British	University	R	2 times at 10 and 20 weeks
SS26	Intervention	51	Male	32.1	White British	College	Y	2 times at 10 and 20 weeks
SS27	Intervention	62	Male	25.5	White British	College	Y	1‐20 weeks
SS28	Control	58	Male	42.5	White British	University	Y	1‐10 weeks
SS29	Control	69	Male	33.5	White British	College	R	1‐10 weeks
SS30	Control	74	Male	57.0	White British	High school	R	1‐10 weeks
SS31	Control	84	Male	19.5	White British	University	R	1‐10 weeks
SS32	Control	62	Male	46.2	White British	University	Y	1‐10 weeks
SS33	Control	64	Male	18.8	White British	University	Y	1‐10 weeks
SS34	Control	68	Male	21.4	White British	Other trade/vocation qualification	Y	1‐20 weeks
SS35	Control	43	Female	15.7	White British	High school	Y	1‐20 weeks

a eGFR: estimated glomerular filtration rate.

b SS: semistructured.

c Y: yes.

d R: retired.

e NR: not reported.

f SE: self-employed.

### Themes

#### Overview of Findings

**Figure 1.**
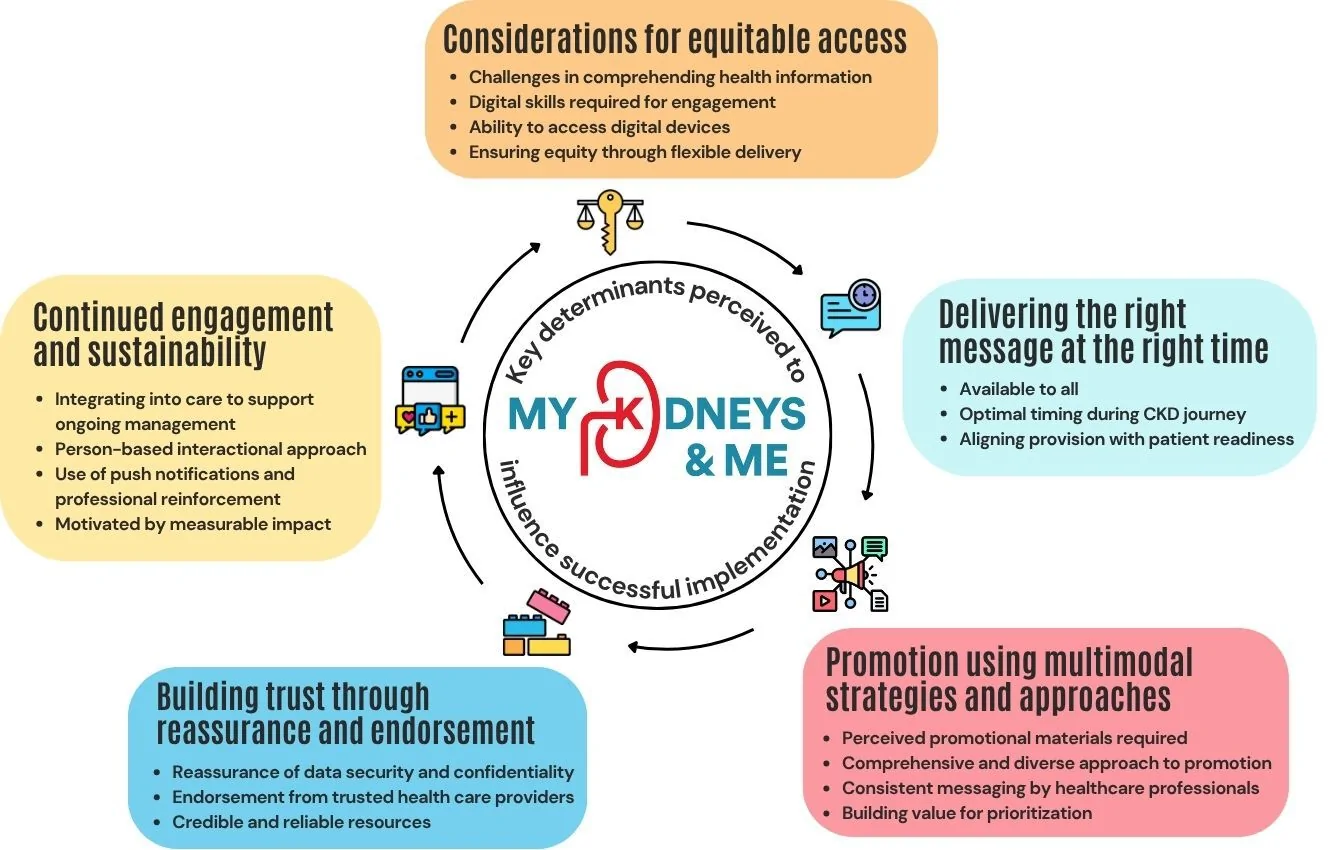
Key determinants perceived to influence implementation of My Kidneys & Me. CKD: chronic kidney disease.

Five overarching themes were identified, each containing subthemes. Many of the identified factors perceived to influence the implementation and sustainability of MK&M DHI could be considered as both a barrier and a facilitator, depending on the context and individual circumstances. These factors can be encapsulated within implementation frameworks as determinants that influence the outcome of implementation efforts and will need to be considered when implementation planning. The key determinants perceived to influence the implementation of MK&M into routine kidney care are shown in Figure 1. The determinants identified are conceptualized as overlapping and dynamically interrelated rather than independent, reflecting the complex ways in which factors influencing DHI implementation interact.

#### Delivering the Right Message at the Right Time

##### Available to All

Participants expressed that the intervention should be widely accessible and routinely available as part of standard care. They emphasized the importance of increasing general awareness and providing clear, comprehensive information to explain the program effectively. Making the resource broadly available could help empower individuals to better manage their health and support their overall well-being.

*I think it would be good to have it routinely available, because I think people have got to the stage, these days especially, where they think oh I don’t want to phone my doctor about that, oh I don’t know if I should be doing this, and I think it would be helpful to be able to look it up and have a bit of a definite answer*.

[SS17 (F, 69, I)]

*I think anything that’s out there that people can just access online and read it, it’s a good thing, as I say, there’s loads of information in the programmes for people...It’s going to help them to manage the condition and to hopefully slow down the progression, I mean, there’s no cure for kidney disease and it is a progressive illness, but...you can slow it down*.

[SS18 (M, 60, I)]

##### Optimal Timing During CKD Journey

There were mixed views on the most appropriate timing for receiving the MK&M DHI. Some participants felt that offering it immediately at diagnosis would be most beneficial, providing clarity and support during an overwhelming period. However, other participants reported preferring a short time period between diagnosis and receiving MK&M, allowing individuals time to process and come to terms with their diagnosis before engaging with additional information. The appropriate time frame suggested for providing MK&M ranged from a few weeks after diagnosis to several months. Despite these differing perspectives, there was a shared consensus that MK&M should be provided early in a person’s CKD journey, enabling individuals to understand their condition and learn how best to manage it.

*It would have been a useful thing at the point of diagnosis. I think that would have really helped actually because it would have sent me away with something...so that at that point, the information about managing the condition and what you can do to potentially arrest its progression is really incredibly useful*.

[SS01 (F, 38, I)]

*I suppose by the time you’ve got a consultant, you’ve actually got a proper diagnosis...you’ve gone to see them initially and then they’ve given you the bad news, and—I think maybe before you go see them, oh here you go, you can have a look at this—maybe it’s a bit too daunting...Maybe to see them first, so you’ve got some rapport going. And then if you did have any issues or questions or what have you, you could talk to them after that*.

[SS06 (F, 55, I)]

*I think it’s better to be proactive really and I would think that something like the My Kidneys programme should probably be broadcast more widely...I think because there was a gap between when I was diagnosed and treatment was planned, it might be attractive to offer it at diagnosis, on the other hand of course some people might be in a bit of a state...So it might be better if it’s just slightly after the diagnosis when people have had a chance to think, you know, what am I going to do*.

[SS30 (M, 74, C)]

##### Aligning Provision With Patient Readiness

The timing at which MK&M is provided and how it is communicated were considered to be crucial by participants and may require some flexibility to accommodate individual readiness. Participants suggested that there could be a most effective moment for providing MK&M, but this would depend on when an individual is most receptive to learning and motivated to engage with information about their CKD. Participants explained how clear, empathetic communication and repeated opportunities are essential to ensure that individuals receive MK&M when they are prepared to act, ultimately enhancing engagement and self-management.

*It’s initially quite a lot to take on. You think oh my goodness. But if people can be brought round to the idea of well it is a new way of life because that’s what you want, isn’t it, you do want life, you don’t want to not have life*.

[SS17 (F, 69, I)]

*I think maybe thinking about people’s feelings, maybe it shouldn’t be right at the minute you get diagnosed, maybe it should—you perhaps need that to sink in a little bit first—I think whenever you're given any sort of diagnosis it’s something you need to come to terms with first of all*.

[SS18 (M, 60, I)]

*Having something online like that would actually I think be very beneficial. when patients are first diagnosed that’s when they want to know all the information about when they're going to help themselves and do this and do that*.

[SS05 (F, 61, I)]

*It’s about accessibility, isn't it, about context and benefits that an individual receives from that and all that will always be an individual situation, so then you can look at it in the broader sense it will always be down to the individual as to how that’s perceived isn't it*.

[SS02 (F, 66, I)]

### Promotion Using Multimodal Strategies and Approaches

#### Perceived Promotional Materials Required

Participants suggested practical ways to promote access to MK&M, including a leaflet with a QR code linking directly to the resource. They felt that this should ideally be provided by a clinician during an appointment to reinforce trust and encourage engagement. Posters with QR codes in waiting rooms were also recommended as a visible and convenient option. In addition, participants proposed sending information via post, email, or SMS text message, ensuring that those who do not have regular appointments can still easily receive information and guidance about how to access MK&M.

*Possibly a leaflet or a poster in the waiting room type thing with a QR code or something like that*.

[SS08 (M, 54, I)]

*I think a flier is always helpful to people, they can come away and think about it...most people have an email address so there’s potential either to do that online or the piece of paper*.

[SS02 (F, 66, I)]

*Have a leaflet. Hand it over. Put a QR code on the leaflet and tie it to the app so it’s easy to download...If your clinician could pass over a leaflet at the point of an appointment then I think that would be a good thing*.

[SS01 (F, 38, I)]

#### Comprehensive and Diverse Approach to Promotion

Both general practitioner (GP) practices and hospitals were seen, by participants, as responsible for distributing promotional materials, ensuring consistent communication and availability across care settings. Many felt that the information should ideally be provided by a clinician during an appointment, followed by additional information later to act as a reminder. Multiple touchpoints were considered important because patients may not act on information the first time it is offered, and repeated exposure was felt likely to increase awareness and facilitate uptake and ongoing use.

*Electronically through email...that’s probably the easiest thing, or through a text even, I mean, people have got, probably got, most people have got phones haven't they. Or face to face, it doesn’t matter. Actually, my preferred method would be probably through a professional! Because then I’d trust it basically*.

[SS32 (M, 62, C)]

*I always like a face to face with a letter follow up. Because I think the face to face, well I like face to face so I can ask questions and I like the follow up letter for the things that I might have forgotten after being told whatever’s wrong with me. I prefer a letter. I just like the consultant’s letters, you know, it’s set out just exactly what’s wrong and what you’ve discussed. If there’s any leaflets on the subject I don’t mind those being included*.

[SS34 (M, 64, C)]

*A leaflet handed to them saying if you have got access to it look at this, look at that or best practice, these are the dos and these are the don’ts. And maybe the doctor could hand one of those leaflets out...If it is not the doctor then the nephrology clinic should hand them out...I’d like it in a letter form first of all rather than a random e-mail*.

[SS29 (M, 69, C)]

#### Consistent Messaging by Health Care Professionals

Promotion of MK&M by doctors or other members of the multidisciplinary team at clinical appointments was believed to encourage engagement. Participants felt that all health care professionals, across GP practices and hospital settings, should actively advertise and promote MK&M, ensuring consistent messaging throughout the patient journey. Having different members of the multidisciplinary team reiterate the message and provide reminders of MK&M at multiple points, using the same information and consistent style of delivery, was deemed important. Repeated and uniform messaging was seen as essential for encouraging engagement and preventing the information from being overlooked. This approach was perceived to normalize MK&M as part of routine care and increase the likelihood of patients engaging with it.

*I think it’s important to come from a professional and I think it should be common across the professionals so they give the same information as well. And the same style of delivery if possible*.

[SS34 (M, 64, C)]

*Nurses mentioning it or giving you leaflets or you can collect some sort of a booklet from somewhere when you go for tests, etc. Or to see a doctor...when you go for your blood pressure tests I guess the nurse could say something about it or whatever if they were more informed.*

[SS24 (M, 72, I)]

*The obvious things through GPs and nephrology units in hospitals and medical centres,...I think they could easily offer it in much the same way that it was offered via my consultant, I mean, the GP could have said, knowing I’ve got this long term kidney problem, why don’t you try this programme because I think it might help you.*

[SS30 (M, 74, C)]

#### Building Value for Prioritization

Participants believed that for individuals to benefit from MK&M, they need to perceive it as an opportunity to actively engage and take greater control of their health and well-being. Dedicating time to understanding the information provided by MK&M and applying these insights was believed to help people make informed decisions, manage their health more effectively, and feel empowered in their everyday lives. To ensure that MK&M becomes a priority for patients, participants considered it essential to create a personal connection to the program and provide support that encourages proactive steps toward better self-management. Participants also highlighted the need to actively promote and “sell” the value of MK&M, so that individuals clearly see its benefits and feel motivated to invest their time and effort.

*Hopefully it’s self-interest. I can't think of any other reason you would need, you would want to help, you should want to help yourself.*

[SS25 (M, 76, C)]

*You need to tempt it—and it sounds ridiculous...but whatever it is you need to sell it...I think people are just so kind of perhaps naive or whatever, but it just needs to be sold to them, whatever it is, to get their interest*.

[SS07 (M, 55, I)]

*I suppose life gets in the way, other things get in the way, you get waylaid by things...But I think the more informed you can be and I see this platform as giving me information and helping me take control, which is what I’m looking forward to really. Quite nice to have that guidance.*

[SS13 (F, 57, I)]

*You need to be able to engender some sort of personal attachment to it really don’t you...I don’t know how you get people to take onboard their own authority in engaging and taking on some individual thought process!...I don’t know how do you get people to start to think about themselves. You see you’ve got me more engaged now because I feel a sense of personal involvement to a degree.*

[SS17 (F, 69, I)]

### Considerations for Equitable Access

#### Challenges in Comprehending Health Information

Health literacy was identified as a potential barrier to engaging with MK&M. Participants described how many individuals lack awareness of how best to look after their health and may not find the program useful if they do not understand the information provided. Participants, who considered themselves to be well educated, reported difficulties pronouncing medical terms, describing them as “another language” that can be challenging to comprehend. They felt that people with low levels of education would struggle, resulting in disengagement. Participants suggested that providing audio support alongside written information, particularly for complex terminology, combined with clear explanations, could significantly improve health literacy and enhance engagement with MK&M.

*It sounds ridiculous, but trying to be less medical, OK, to put your head inside the patient who’s going to know nothing about it whatsoever...but without being patronising*.

(SS08 (M, 55, I)]

*Illiteracy levels. People are not aware of how they can help themselves, I think that’s part of the problem*.

(SS13 (F, 57, I)]

*There are certain words on it that you can't really pronounce and if you're not literate, you know what I mean, it does affect you, or if you're not... you know, if you’ve not got that brilliant education*.

(SS10 (F, 60, I)]

#### Digital Skills Required for Engagement

Participants discussed how individuals with low levels of digital literacy may face challenges in accessing and engaging with MK&M. To overcome this barrier, they suggested having “digital buddies” or “digital champions”; supportive individuals who can help participants develop essential digital skills and build confidence in using technology to promote inclusion and ensure that everyone can benefit from MK&M. It was considered that the majority of patients would be able to use MK&M, as the digital skills required are not overly complex. Participants noted that if individuals can get online or use an app, they should be able to navigate MK&M successfully.

*You need to be someone who’s either, well who’s happy and confident using either a smart phone, smart device, computer, whatever. The barrier might be maybe more older people who aren’t so happy using something like that. I would have thought most people my age and younger wouldn’t have any great problem with it*.

[SS14 (M, 57, I)]

*I think modern technology is a barrier to a lot of people, particularly older people or people with learning needs. You tend to need to have a certain technical knowledge these days just to survive...a lot of people my age and above aren’t quite as tech savvy as a twenty year old...As we know 90% of people could probably use the programme online*.

[SS18 (M, 60, I)]

*I’m sure that there are people who would struggle with it but I think the vast proportion wouldn’t. I think it’s pretty straightforward*.

[SS25 (M, 76, I)]

*I think there’s always going to be a few people with difficulties. I think mainly because of it is online, but I think in this day and age, ninety something percent of people will be able to get online and get an app or something*.

[SS27 (M, 62, I)]

#### Ability to Access Digital Devices

Digital poverty was identified as a potential barrier to engagement. Participants explained that some individuals may not own a computer or a smartphone, have access to one, or be able to afford a digital device or an internet connection. Participants perceived that there is always a risk of excluding people but believed that most individuals would have access to a digital device. Delivering information digitally was considered the most appropriate approach to ensure widespread availability. Suggestions about how to address gaps in access included creating opportunities for people to come together in group settings or encouraging the use of community resources such as libraries, where access to technology and support may be available.

*If they’ve not got a phone or if you’ve got no credit on your phone or whatever, but you don’t even need credit because it does it…you know, you can do it via your broadband, you know what I mean, you can do it on your laptop or on whatever you set it up on, so the only barrier that I would say is the individual*.

[SS10 (F, 60, I)]

*Certainly using it online or on an app is probably the most sensible way. There is always a danger that it excludes, there are always going to be people that haven’t got phones or what have you, so there’s always a danger of that, but I think realistically it probably is the most sensible way to do it*.

[SS26 (M, 51, I)]

*If you’ve got access to a computer it’s good. I can use it but again we’ve got to think about the people who can’t or can’t afford it or just can’t manage with computers. And I think these days I think you’ve got to think of both things*.

[SS29 (M, 69, C)]

#### Ensuring Equity Through Flexible Delivery

Participants emphasized that accessibility is essential for equitable support. While online resources were seen as useful, many noted that personal circumstances, such as limited internet access, cost, or difficulty using technology, can make DHIs, such as MK&M, impractical for some individuals. They felt that alternative methods of delivering the educational content were needed for those who cannot access the online platform and should be offered in multiple formats. Participants also highlighted that health information and education should be tailored to different learning styles, using audio-visual formats alongside written materials, and supported by face-to-face sessions for clarity and personalized guidance.

*As long as there’s a mixture of - it’s not just all one particular mode of learning, you know, so it’s not just all online or in person or, you know, so a mixture of different things I think I’d prefer then it makes it more real, being able to interact but also do your own work and reflection.*

SS11 (M, 59, I)]

*These computer based sessions are very good if you enjoy doing computer based sessions...Personally, I get a lot more benefit from a face-to-face practical session than from a screen. It’s just the way I like to learn. I get far more from the visual*.

SS12 (M, 72, I)]

*Maybe they would benefit from some sort of pamphlet or something, where it’s just sort of like small concise bits, as opposed to maybe a whole programme*.

SS06 (F, 55, I)]

*If you could condense that programme with just the facts that would really help people, even if it was in a book form or a programme like you're doing...I think a booklet or something would probably benefit people that are starting out on a kidney problem. Then they could have perhaps at the end of that book a contact to go onto something further like this...because that would get them interested in it and then once they're interested in it they’ll be happy to go further*.

[SS01 (F, 38, I)]

### Building Trust Through Reassurance and Endorsement

#### Reassurance of Data Security and Confidentiality

Data protection was a key concern for most participants, who acknowledged that people can be cautious about sharing personal information online. Some individuals reported how they may be particularly suspicious about how their data are collected and used. Participants emphasized the importance of providing clear reassurance about data security, confidentiality, and anonymity to build trust and encourage participation in MK&M and other DHIs.

*I suppose one of the views that you have that’s always in the back of my mind about the data that you say, the things that you say and the information that’s collected, that sort of stuff - need to be shared, so the anonymity is quite good in one sense isn’t it. Because you see all the things about data breaches and all that sort of stuff*.

SS11 (M, 59, I)]

*People don’t like to think people have got information about them and things like that, very wary in this day and age about releasing digital information, like any digital thing people aren’t savvy about keeping their details locked away...You can take all the precautions you like and you can tell people that, whether they want to believe you is another story*.

SS29 (M, 69, C)]

*I don’t want to give data out willy-nilly...I think some sceptics you won’t ever reassure*.

[SS13 (F, 57, I)]

#### Endorsement From Trusted Health Care Providers

Participants emphasized that MK&M should be recommended by a known, trusted health care professional. Having clinician endorsement was seen as critical for credibility and uptake. It was believed that if health care professionals were invested in MK&M and its benefits, patients would be more likely to engage. While MK&M was perceived as potentially available nationally, participants highlighted the importance of providing relevant information locally through GP practices and hospital renal units to strengthen trust and accessibility.

*If a clinician was able to go “You have chronic kidney disease. Your level of function is level 3a” or whatever. “This means that your GFR is x but the likelihood is that you’re going to have this for a long time. Here’s an app that would help you manage this condition”—that would have helped a lot...They’re just authoritative. They’re the person that you trust and if they are invested in the benefits of it you will get patient investment*.

SS01 (F, 38, I)]

*I would definitely use something if it was given as advice to use it I would definitely use it, especially if they came from a trusted source like a doctor or a hospital or whatever, or even somewhere on a trusted source on the internet that you trusted like a charity or something you would go to. I would go to them*.

SS32 (M, 62, C)]

*I feel more assured if it’s come from somebody professional straight out. If it’s from the doctor you tend to believe them more or the nurse practitioners...rather than get the information off a friend or a neighbour*.

SS34 (M, 68, C)]

*If somebody that you trust, a medical professional that you trust is recommending a programme, I think you'd be more likely to do it than if you just kind of googled something*.

[SS35 (F, 43, C)]

#### Credible and Reliable Resources

The reliability of the information provided was considered to be essential. Participants discussed previous difficulties experienced in accessing reputable resources and expressed the need for a central, trusted source where all relevant information is available. A single access platform was perceived to save people from having to search and “dig” through the internet for appropriate content. Having credible resources that avoid speculation and eliminate the risk of harmful or inaccurate information was believed to be critical to supporting patient confidence and engagement in accessing and using digital health information.

*It’s completely reliable, so that’s one of the things that I found really difficult about kidneys in particular...I found [it] really difficult to get information on and it’s just difficult, like even the kidney charities, their information is not so great. So, in terms of a resource about information and treatment and progression of the disease, it’s a reliable source that I can go back to that’s stable*.

SS01 (F, 38, I)]

*I like to go to things that would be trusted, so I want to go to a trusted source not a speculative one. The British Heart Foundation, I would trust them, the national kidney thing, your research thing, I would trust you, you know...I’m very sceptical of some American stuff and YouTube*.

SS32 (M, 62, C)]

*There is a lot of bullshit on the internet so you look for sources that are OK or that you can see instantly that consider to be golden source. Certain charities yes and certain websites absolutely not—I wouldn’t believe a word they say even if it happened to be the truth, it’s surrounded by verbiage and selling opportunities*.

[SS33 (M, 64, C)]

### Continued Engagement and Sustainability

#### Integrating Into Care to Support Ongoing Management

Participants valued the holistic nature of MK&M, as it addressed not only physical health but also psychosocial well-being. To encourage sustained engagement, they felt that it should be closely integrated with their care and used to support the ongoing management of their CKD. Participants believed that health care professionals should adopt an individualized approach when promoting MK&M, signpost to relevant aspects of the program based on health status, and be appropriately followed up.

*The thing that occurred to me was you can really tie this into people’s care. So there are points at which I have to monitor my blood pressure on a weekly basis. So if there was an option that you could flag that says “I’m going to monitor my blood pressure” and it reminds you to do it, that would be really helpful...it would be potentially quite empowering*.

[SS01 (F, 38, I)]

*I guess if something happened that I wasn’t quite sure about or I thought there might be some information to support me on there with it or something I hadn’t taken in or I want to revisit because can’t quite remember what I learned from it in the first place I absolutely would think about going back into it. I don’t feel it’s I’ve ticked all the boxes and that’s it, done, I don’t see it like that, I see it as an additional support trying to work my way through this ‘fun’ situation!*

[SS02 (F, 66, I)]

#### Person-Based Interactional Approach

Participants highlighted the value of face-to-face interaction and how it can complement the digital element of MK&M. Human connection was believed to increase engagement, whether through health care professionals or peer support that fosters a sense of shared experience. Participants felt that it was reassuring to have someone to talk to rather than feeling like communication is solely with a machine. To create a more personal and interactive experience, participants suggested using live chats and Q&A sessions. These options were also seen as a way to build confidence in using the DHI. While general health and disease information could be delivered digitally, it was recommended that personalized information for an individual should be provided in person.

*I think there’s still a place obviously for face to face, just depends on what the situation is, but I think as far as overall help and education is then, yeah, I'm all for online stuff. If it’s just general health education I prefer it online. For something specific to me then I would prefer face to face*.

[SS23 (F, 54, I)]

*You’ve got to make them human and even things like live chats, Q&A live chats or this kind of stuff might be something, just a little bit more human, because otherwise you think you're talking to a machine, which you could be in many ways, you don't bother and you definitely don't interact and you probably don't get the correct answers anyhow...it needs to be slightly more human, because you're talking about people’s lives, their bodies...we all rely too heavily on computers and forget we’re all humans*.

[SS07 (M, 55, I)]

*They’ve got to be backed by manned, where you can actually speak to someone because the problem with the unmanned ones is that they will give you an answer but what they don’t know is if, the nuance isn’t there. If I want to ask about a specific thing or worry about it, they can’t do that. They’re alright as an initial thing but I think that they’ve got to be backed up by an in-person contact, because otherwise you know you’re not really going to be confident about it*.

[SS31 (M, 84, C)]

#### Use of Push Notifications and Professional Reinforcement

Participants appreciated the MK&M platform reminder functions but suggested increasing their frequency. They preferred reminders delivered as push notifications rather than solely via email. Additionally, they believed that health care professionals should play an active role by reinforcing the message and reminding patients about the program. It was felt that this would serve as a memory prompt to maintain engagement, particularly over the long term.

*It’s just that reminder isn’t it, or even just an email to say have you finished the sessions, have you recorded anything this week, have you done this—just a memory job because once you’ve done the reading and you come out of it and busy you just—I just literally forgot about it. So, it I had an email dropping in saying ‘have you thought about logging this or have you done this or visit the chat room’, or something, I think it might have probably made me log back in again*.

SS23 (F, 54, I)]

*Like a monthly reminder email have you visited the thing or something that would help, because the world’s full of good intentions and you try to do things and if you just got a reminder once a month, you know, pretty much like if you get an email...just to say have you checked out this topic*.

SS27 (M, 62, I)]

*Getting the boosters to remind you to do it I think! That probably is a good reminder because I think we all, sort of forget and you think oh I’ve done it now because I’ve read it, maybe need to go back and look at it again, so that is a good idea*.

[SS05 (F, 61, I)]

#### Motivated by Measurable Impact

Observing positive changes and experiencing benefits from engaging with MK&M were reported as strong motivators for continued participation. Participants highlighted the importance of committing time to improving health and making appropriate lifestyle changes. The MK&M DHI was valued for its ability to allow users to pick up where they left off, monitor progress, and track achievements over time. Features such as goal setting were seen as particularly motivating, as successfully reaching goals encouraged individuals to set and achieve new ones, reinforcing ongoing engagement.

*You can see how far you’ve got, for a start. And it’s nice to know you can carry on from where you’ve left off. You’ve got these bits on the dashboard which are quite good, it tells you how far you’ve got, and what have you...I can see some changes or something, so maybe when I get to the booster sessions I can set some goals and what have you, then maybe if I see some changes then I’ll be more—yeah, let’s do this, you know, more often*.

[SS06 (F, 55, I)]

*I think if you’re interested, and I am, I’m always interested in health, not as an egocentric thing, just because we want to live the best that we can. I want to make sure I get the best out of life by making sure that I’m ready to face the challenges. So I think this looks really interesting. The more I look at it the more I see that there’s there, so bring it on*.

[SS13 (F, 57, I)]

## Discussion

### Principal Findings

This qualitative substudy explored SMILE-K trial participants’ perspectives about the implementation and sustainability of the MK&M DHI to understand how patients would like MK&M to be delivered in clinical practice. The study findings provide a detailed account of factors patients felt were important to consider to support the implementation of MK&M into routine patient care to ensure that it meets real-world needs. This information offers critical context and is being used to inform the development and testing of implementation strategies, ensuring that MK&M is relevant, acceptable, and feasible in real-world practice.

The perceived most appropriate timing for MK&M to be provided was highly variable, with suggestions ranging from at diagnosis to a few weeks or several months later. This reflects the need for flexibility in delivery or prescription of MK&M and should be based on individual readiness rather than a particular time point in the patient’s CKD journey. While there may not be a single teachable moment in the CKD journey, identifying a moment where individuals are more receptive to health messages, which may not necessarily correspond with a health event, will increase the likelihood of a resulting behavior change [21,22]. This underscores the importance of revisiting the offer multiple times throughout the early stages of the journey, as initial discussions may not always lead to uptake. Providing repeated exposure and opportunities can increase awareness and uptake of MK&M. It aligns with patients’ desire for flexibility and autonomy, ensuring that they can engage with MK&M when they feel ready and motivated to act. Considering the COM-B (Capability-Opportunity-Motivation Behavior) model [23] to determine patients’ readiness and receptivity to interventions, such as MK&M, may offer opportune intervention points, and capability or opportunity barriers may preclude behavior change that might be expected based on motivational shifts [24]. Understanding an individual’s specific barriers and strengths can enable tailored strategies to increase acceptance and engagement, ultimately enhancing self-management.

Key barriers identified to accessing and engaging with MK&M included low health and digital literacy, as well as digital poverty. These barriers reflect broader challenges in CKD education, where low awareness of the condition and its risks [25], limited health [26,27] and digital literacy [28], and a lack of accessible educational materials for at-risk populations [29] are well-documented obstacles to effective patient engagement. Such challenges are not unique to CKD and similarly affect the management of other long-term conditions [30], including diabetes [31] and chronic obstructive pulmonary disease [32], and musculoskeletal conditions [33]. Clear communication from health care professionals, combined with patient reinforcement and targeted education, plays a critical role in improving health literacy and promoting effective self-management [34-36]. Delivering health messages in a clear, concise manner, using simple language and avoiding medical jargon, is essential to promote understanding, especially in those with limited health literacy [36]. Our previous work identified that while patients found the information provided in MK&M to be comprehensive, its presentation made it easy to access and engage with [13]. Despite positive feedback, it is clear from this study that further refinements are required to MK&M and the delivery of the education to ensure equitable access to all people living with early-stage CKD, including those from disadvantaged and underserved groups. Offering content in multiple formats (eg, print, video, and audio) and providing tailored support options can help overcome barriers faced by individuals experiencing digital poverty or low digital literacy. Future work should focus on enhancing DHIs, such as MK&M, to improve inclusion for individuals who are potentially disadvantaged yet stand to benefit most, with efforts aimed at addressing these gaps and ensuring equitable access.

Participants felt that interventions endorsed by trusted health care professionals and delivered with consistent, unified messaging are more likely to build patient confidence, encourage engagement, and improve adherence. This highlights the importance of health care professional involvement and consistent messaging in driving patient engagement, as interventions lacking such endorsement may struggle to achieve widespread adoption. Research examining patient portals found that most interventions were not delivered by a health care provider or an administrator [37], which may limit uptake of such tools by patients who may need encouragement and support to access these resources [38]. Health care providers may need to shift from acting as intermediaries, controlling and relaying information, to serving as apomediaries (ie, people who help users navigate information independently), guiding patients toward high-quality information and services [39]. MK&M offers a potential solution to facilitate clinical care by providing comprehensive, evidence-based, and disease-specific information and supporting ongoing disease management. It can be integrated into clinical appointments and tailored to individual patient needs, which may enhance engagement and the health care professional–patient relationship [40]—a key factor in effective CKD self-management [7].

Integration of MK&M into clinical care and using it to support the ongoing CKD management was believed to be vital to encourage sustainability. The COVID-19 pandemic accelerated the uptake of digital tools and platforms within the National Health Service [41], leading to substantial transformations in service delivery [42]. Central to the National Health Service 10-year plan is the adoption of technology-enabled solutions, transforming traditional models into digitally connected, patient-centered care [43]. Digital health ecosystems have the potential to build long-term, collaborative relationships between patients and care teams that encourage and support maintenance of health-promoting behaviors [44]. However, in order for DHIs to deliver maximum impact, they must be effectively adopted and embedded into routine practice. The introduction of Integrated Care Systems and virtual care programs for CKD, such as LUCID [45], that are designed to optimize CKD management, provides a promising solution for integrating MK&M into routine care. Embedding MK&M within these models could strengthen patient education and awareness, supporting more comprehensive and coordinated care.

Assessing the readiness of health services to integrate these technologies is critical to identify gaps, allocate resources, and enable successful implementation and sustainability. Implementation models and frameworks can be used to systematically identify and map potential barriers and facilitators, link them to appropriate strategies, and guide the development of comprehensive implementation and evaluation plans. Jassemi and colleagues [38] illustrate this through the integration of the patient-focused eHealth intervention, My Kidneys My Health, into primary care and general nephrology kidney care. Key barriers identified by health care professionals included competing priorities and patient accessibility challenges (eg, internet access, technology use, and health literacy), while enablers included the ability to tailor the intervention to supplement education and alignment with core values such as accessibility, credibility, and usefulness [38]. These factors mirror those reported by patients in our study, particularly around digital literacy, engagement challenges, and the need for organizational support. This highlights the shared importance of usability, accessibility, and perceived value in successful implementation. At the same time, our data add patient-specific insights on autonomy, personalized communication, and uncertainties about how the DHI would fit within existing care pathways, and in some areas diverge from professional assumptions (eg, differing expectations about usability and required support). Together, these complementary and contrasting insights offer a more comprehensive understanding of factors shaping DHI implementation. Ensuring that interventions meet both clinical priorities and patient needs can increase the likelihood of successful adoption and sustained engagement. Future work should focus on co-designing implementation plans and readiness assessments to strengthen alignment of perspectives and enable scalable, sustainable implementation of DHIs to support CKD self-management.

Across long-term conditions, the value of DHIs is evident; however, their effectiveness and implementation challenges differ markedly between conditions and remain dependent on successful integration into routine care workflows. Successful implementation is consistently linked to patient-centered design and attention to individual, technological, and organizational factors that shape real-world use [46,47]. DHIs are most effective when they offer personalization, clear feedback, and simplicity, with human connection (eg, coaching or guided support) playing a key role in sustaining engagement over time [48]. Although DHIs can enhance self-management and social participation, their effectiveness differs by delivery mode and the degree to which they facilitate meaningful, tailored interaction [49]. Persistent barriers, including limited digital literacy, variable user confidence, and usability or access challenges, especially among older adults and people with multimorbidity [50], continue to hinder adoption. In addition, poor integration with existing workflows and clinical record systems constrains scalability and increases workload for staff, further limiting sustained implementation in routine practice [51]. Even structured implementation support may not overcome these challenges. A cluster randomized controlled trial of an embedding package for diabetes self-management education reported limited clinical improvement and difficulties normalizing new practices in primary care [52]. Primary care teams struggled to embed self-management education into routine practice, with low referral and attendance, no meaningful clinical improvement, and persistent difficulties normalizing new workflows, despite structured support. Competing clinical demands, entrenched routines, and limited prioritization of self-management contributed to poor uptake, alongside ongoing inequities in engagement across patient groups. These findings reinforce that implementing MK&M successfully will require not only robust technological functionality but also seamless integration into routine care and targeted strategies to address digital and health literacy, ensuring that the DHI remains relevant, acceptable, and feasible in real-world practice [53].

### Strengths and Limitations

This study has several strengths and limitations. A key strength of this study was the use of qualitative methodology, which enabled us to obtain rich, in-depth insights into participants’ experiences and perspectives, providing a nuanced understanding of factors that will affect the implementation of MK&M. The inclusion of participants from both the intervention group and the control group allows for exploration of differences across groups. Although participants had not used the DHI themselves, their organizational and experiential insights provide an important perspective on factors that shape adoption, readiness, and feasibility. These perspectives therefore make a meaningful contribution to understanding the wider implementation context within which the DHI will operate. It also offers context for perceived benefits and challenges and provides an insight into how implementation factors (eg, usability and accessibility) influence engagement. This approach supports patient-centered learning that can inform future design and implementation strategies. Future work will build on these insights to test implementation strategies in real-world settings, ensuring scalability and sustainability. Implementation models and frameworks were not included in this exploratory study to enable the identification of context-specific issues that may not be fully captured by existing frameworks. However, the findings will inform future DHI implementation strategies with implementation plans to be supported and adapted using an appropriate framework.

A limitation of the study was the potential bias of participants recruited, which may limit the generalizability of the findings. While efforts were made to select a variety of participants, using maximum variation sampling, there was self-selection bias as some participants who were invited to take part did not respond to the invitation. Some subthemes, such as digital poverty, reflect the experiences of underrepresented groups who were largely absent from the sample, potentially limiting the depth of insight in these areas. Despite this, most participants still recognized and discussed issues relating to digital poverty, even if they had not experienced these challenges themselves. As the views represented in our study may not fully capture the experiences or barriers faced by underrepresented or digitally marginalized populations, the generalizability of these specific themes is constrained, and our findings should be interpreted with this context in mind.

Delivering the trial and intervention virtually created barriers for individuals with poor digital literacy and rendered those without digital access ineligible. As a result, valuable insights from participants who may face the greatest barriers to engagement were missed. Future research that actively incorporates the perspectives of disadvantaged populations is essential to ensuring that interventions and policy recommendations address the full spectrum of barriers and support greater equity in digital health engagement. Their perspectives could inform strategies to improve accessibility, usability, and equity. Thus, future research should include enhanced strategies to actively include digitally underserved populations through alternative recruitment and multiformat delivery, addressing digital poverty and low literacy to ensure inclusive, scalable, and sustainable implementation.

Participant experiences were collected within the context of a trial. Although the trial took a pragmatic approach and aimed to reflect aspects of routine practice, providing less structured support than traditional trials and allowing participants to use the MK&M DHI as they wished, elements of the trial environment may still have fostered more positive views of sustainability, feasibility, and integration than might be observed in everyday clinical settings. Trial features, such as clearer processes and greater predictability, can create a more enabling context than routine care, where competing demands and resource constraints are more prominent. As such, the influence of the trial context should be considered when interpreting the transferability of these findings to real-world implementation.

### Conclusions

Our study highlights that timely, well-targeted communication using diverse strategies is critical for the successful uptake of MK&M among people living with CKD. The identification of perceived factors that will affect implementation of the program provides actionable insights to guide the development of tailored implementation strategies. These findings highlight the need to align MK&M with real-world contexts to ensure that it is relevant, acceptable, and feasible in routine practice. Consideration for the multiple contextual factors required, and the equity challenges, in integrating MK&M into routine kidney care, is essential for implementation to be successful. Embedding these principles into implementation planning and addressing key contextual factors can enhance the quality, effectiveness, and sustainability of kidney care interventions, such as MK&M, ultimately supporting patient care and improving outcomes.

## Supplementary material

10.2196/91966 Checklist 1 COREQ checklist.

## Abbreviations

COM-B: Capability-Opportunity-Motivation Behavior
DHI: digital health intervention
eGFR: estimated glomerular filtration rate
GP: general practitioner
MK&M: My Kidneys & Me
